# Patagonian Fjords/Channels vs. Open Ocean: Phytoplankton Molecular Diversity on Southern Chilean Coast

**DOI:** 10.3390/microorganisms13122746

**Published:** 2025-12-02

**Authors:** Gonzalo Fuenzalida, Roland Sanchez, Andrea X. Silva, Alvaro Figueroa, Osvaldo Artal, Maria Fernanda Torres, Alejandro E. Montecinos, Milko Jorquera, Nicole Trefault, Oscar Espinoza-González, Leonardo Guzman

**Affiliations:** 1 Departamento de Ciencias Básicas, Facultad de Ciencias, Universidad Santo Tomas, Av. Ramon Picarte #1160, Valdivia 5090000, Chile; 2 Centro de Estudios de Algas Nocivas (CREAN), Instituto de Fomento Pesquero, Puerto Montt 5500000, Chile; oscar.espinoza@ifop.cl (O.E.-G.); leonardo.guzman@ifop.cl (L.G.); 3 Dirección de Investigación sede Puerto Montt, Universidad Austral de Chile, Puerto Montt 5480000, Chile; roland.sanchez@uach.cl; 4 AUSTRAL-*omics*, Vicerrectoría de Investigación, Desarrollo y Creación Artística, Universidad Austral de Chile, Valdivia 5110566, Chile; andrea.silva@uach.cl (A.X.S.); alvaroalejandrofigueroa@gmail.com (A.F.); 5 Geodel Laboratory, Departamento de Ingeniería en Obras Civiles, Facultad de Ingeniería y Ciencias, Universidad de la Frontera, Temuco 4810296, Chile; osvaldo.artal@ufrontera.cl; 6 Center for Oceanographic Research COPAS Coastal, Universidad de Concepción, Concepción 4070386, Chile; 7 Instituto de Ciencias Ambientales y Evolutivas, Facultad de Ciencias, Universidad Austral de Chile, Valdivia 5110566, Chile; fertorresc@gmail.com (M.F.T.); jano.montecinos@gmail.com (A.E.M.); 8 Laboratorio de Ecología Microbiana Aplicada, Departamento de Ciencias Químicas y Recursos Naturales, Scientific and Biotechnological Bioresource Nucleus (BIOREN-UFRO), Universidad de La Frontera, Temuco 4810296, Chile; milko.jorquera@ufrontera.cl; 9 Centro GEMA—Genómica, Ecología y Medio Ambiente, Facultad de Ciencias, Universidad Mayor, Santiago 8580745, Chile; ntrefault@gmail.com

**Keywords:** SSU rRNA, metabarcoding, phytoplankton diversity, harmful algal blooms, fjords, Southeast Pacific, environmental filtering, biogeography

## Abstract

Environmental filtering studies have revealed immense oceanic microbial diversity, yet the Southeast Pacific remains comparatively undersampled. We characterize the molecular diversity of phytoplankton across two biogeographic domains with contrasting oceanography—fjords and channels (41–53° S) versus the open Pacific (36–42° S)—where the frequency and intensity of harmful algal blooms (HABs) have increased. Using SSU rRNA metabarcoding, we retrieved community composition and biogeographic patterns for micro-phytoplankton. Diversity signals indicated broadly overlapping communities between domains with subtle shifts along hydrographic and nutrient gradients rather than sharp breaks. Phylogenetic resolution within bloom-forming genera recovered well-supported clades, and multiple ASVs matched historically relevant HAB taxa, including representatives of the *Alexandrium* complex, *Dinophysis*, *Pseudo-nitzschia*, and *Karenia*. Together, these results suggest that regional environmental filtering acts modestly at the community level while preserving clear signals of taxa of management concern. By providing a regionally resolved, DNA-based baseline for southern Chile’s fjords and adjacent open coast, this study helps fill the molecular diversity gap for the Southeast Pacific and supports improved HAB surveillance and ecosystem forecasting in a climate-sensitive seascape.

## 1. Introduction

Phytoplankton plays a fundamental role in sustaining marine food webs, regulating biogeochemical cycles, and driving ecosystem productivity [1,2]. Harmful algal blooms (HABs), commonly known as red tides, are a striking manifestation of this diversity, resulting from the exponential growth of microalgae in aquatic ecosystems [3]. These events have been recurrent on the Chilean coast, covering extensive geographical regions with ecological, social, and economic consequences, and are primarily caused by dinoflagellates and diatoms from Dinophyceae and Bacillariophyceae classes [4,5,6,7,8,9,10]. Environmental DNA studies (eDNA) have delved deeper into microbial diversity and biogeography across the oceans [11,12,13,14,15], yet the Southeast Pacific remains underrepresented. In particular, the fjord systems of southern Chile are recognized as sentinel areas for climate change research due to their sensitivity to environmental shifts, and they are also key sites for the country’s aquaculture industry [16,17]. Understanding the environmental drivers that shape phytoplankton distribution in this region is critical for predicting ecosystem responses to climate change and for sustaining both biodiversity and economic activities [1].

The southern Chilean coast (36° to 56° S) generates contrasting biogeographic patterns of marine species distribution, shaped by climatic, geomorphological, and oceanographic factors [18]. While the coast between 36° and 41° S is exposed to the open Pacific Ocean, the region south of Reloncavi fjord (42° S) is dominated by fjords and channels formed by tectonics and glaciations, creating highly heterogeneous hydrodynamic conditions [19]. These contrasting settings influence phytoplankton composition, including species responsible for HAB events [4,5,6]. However, high-throughput sequencing (HTS) studies covering this broad latitudinal range are rare.

For southern fjords, HTS work has reported high taxonomic diversity but weak spatial differentiation in diatoms and dinoflagellates, likely linked to estuarine circulation and community homogenization [20,21]. Much HTS work in Chile has focused on specific microbial groups or open-coast sites, leaving Patagonian fjords comparatively underexplored [22,23]. Because these fjords are both sensitive to environmental forcing and central to aquaculture, regionally resolved molecular baselines are needed. Although eDNA/HTS has advanced global maps of marine microbial diversity and biogeography [11,24,25,26,27], the Southeast Pacific—particularly subantarctic fjords—remains underrepresented. In parallel, satellite-based assessments suggest shifting contributions of major phytoplankton functional types at high latitudes and in coastal domains, implying that fjord systems may either amplify or buffer large-scale trajectories. Together, these local contrasts and global signals underscore the urgency of documenting phytoplankton biogeography with approaches that capture cryptic and low-abundance taxa, providing the context for a targeted, region-wide baseline across fjord and open-coast domains.

We addressed this gap by applying eDNA metabarcoding of the 18S rRNA V4 region (primers TAReuk454FWD1/TAReukREV3) within a long-term HAB monitoring program spanning ~36–42° S. Spatially structured sampling across two contrasting marine settings—an exposed Pacific margin and a fjord-dominated domain—enabled us to assess how hydrographic context shapes phytoplankton assemblages composition. High-throughput sequencing provides greater taxonomic resolution and sensitivity than microscopy, detecting morphologically cryptic and low-abundance taxa and allowing comprehensive spatial comparisons. We hypothesized that phytoplankton diversity and taxonomic composition would differ between these environments as a function of distinct environmental drivers. Accordingly, we analyzed samples from multiple monitoring stations to generate assemblage profiles and link patterns to bloom-forming groups of management concern. Our overarching objective was to provide a molecular baseline of phytoplankton community composition and diversity patterns across provinces, with emphasis—but not exclusivity—on HAB-linked lineages. By combining metabarcoding with an operational monitoring network, our study delivers a regional baseline of phytoplankton diversity for southern Chile that supports HAB surveillance, improves the capacity to anticipate ecosystem responses in a climate-sensitive seascape, and helps fill the documented diversity gap for the Southeast Pacific, particularly its subantarctic fjord systems.

## 2. Methods

### 2.1. Sampling and DNA Extraction

**Figure 1:**
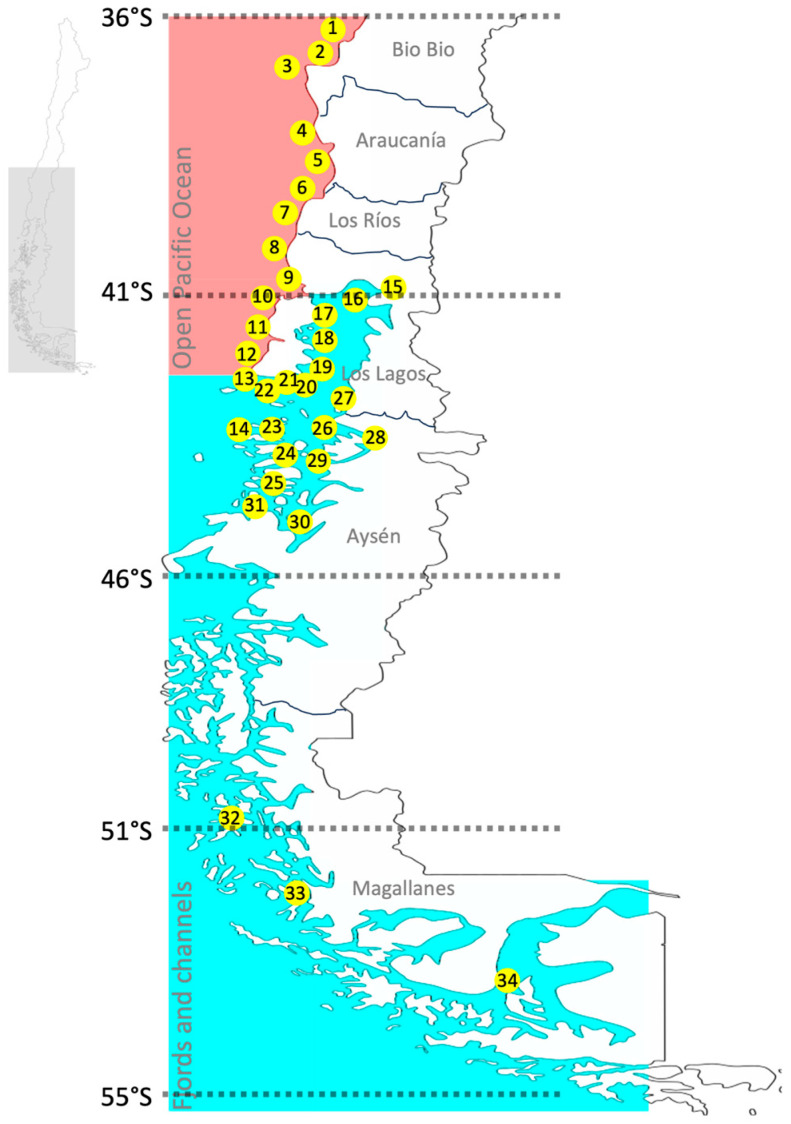
MapMap of the 34 sampling stations (numbered 1–34) distributed in two geographical areas: Open Pacific, stations 1–14 (light red symbols), and Fjords/Channels, stations 15–34 (light blue symbols). The inset map in the upper left shows Chile, where the light gray area indicates the region of the country where the study was conducted. Names correspond to Chilean administrative regions.

To describe phytoplanktonic molecular diversity between open ocean and fjord/channel communities, we implemented a metabarcoding approach amplifying the V4 hypervariable region of the SSU ribosomal gene [28] in 201 samples collected between September 2020 and February 2021, taken from 34 sampling points in the Chilean HABs monitoring program (https://www.ifop.cl/) (Figure 1 and Supplementary Data). The samples were collected from each site using a phytoplankton net (23 μm pore size) and filtered and processed for DNA extraction by the CTAB method. The quality and concentration of the extracted gDNA was verified by Qubit 4 fluorometer (Invitrogen). DNA was stored at −20 °C for further analysis.

### 2.2. Amplification and Sequencing

Two-step PCR amplification was performed for Illumina paired-end library preparation. The first amplicon PCR was performed using eukaryotic universal primers TAReukFWD1 (5′-CCAGCASCYGCGGTAATTCC-3′) and TAReukREV3 (5′-ACTTTCGTTCTTGATYRA-3′) [28] in a total volume of 25 μL, including 5 ng of DNA template, 5 μL each of the forward and reverse primers (1 μM) and 12.5 2× KAPA HiFi HotStart ReadyMix (Roche Sequencing and Life Science). The thermal cycling profile was 95 °C for 3 min; 25 cycles at 95 °C for 30 s, annealing temperature of 55 °C for 30 s, and 72 °C for 30 s; followed by 72 °C for 5 min. The second PCR was used to add Illumina adaptor and indexes to samples. The second PCR volume was 50 μL, including 5 μL DNA of the first PCR, 5 μL each of Nextera XT Index Primers, 25 μL of 2× KAPA HiFi HotStart ReadyMix and 10 μL PCR grade water. The thermal cycling profile was 95 °C for 3 min; 25 cycles at 95 °C for 30 s, annealing temperature of 55 °C for 30 s, and 72 °C for 30 s; followed by 72 °C for 5 min.

The libraries’ DNA concentrations were checked using a Qubit dsDNA HS Assay Kit (Thermo Fisher Scientific, Waltham, MA, USA) and a Qubit Fluorometer (Thermo Fisher Scientific). The libraries were sequenced on a MiSeq system (Illumina Inc., San Diego, CA, USA) in the core facility AUSTRAL-*omics*, Valdivia-Chile for paired-end 2 × 300 bp reads. To reduce the bias effects of sequencing depth on each sample, the expected number of total reads per sample was set to 100,000.

### 2.3. Bioinformatic Analysis

The quality of the libraries obtained was evaluated using FastQC v0.9.11 software, while the readings associated with each sample were separated using the Fqgrep script (https://github.com/indraniel/fqgrep, accessed on 1 January 2024). Nucleotides representing primers, indexes and spacers of each read were removed using Cutadapt [29]. DADA2 [30] and were used to construct ASVs (amplicon sequence variant) with the denoise-paired mode and the following parameters: --p-trunc-len-f 0; --p-trunc-len-r 200; --p-max-ee-f 2; --p-max-ee-r 2; --p-n-reads-learn 1,000,000; --p-chimera-method pooled). The taxonomic assignment of the representative nucleotide sequences of each ASV was performed through the qiime feature-classifier classify-consensus-vsearch command implemented in QIIME2 Software (v2024.10) [31]. PR2 v4.13.0 [32] was used as a reference database with an identity percentage of 90%. Once this taxonomic assignment was obtained, those ASVs classified as Bacteria, Unassigned, Rhodophyta, and Opisthokonta were removed. Rarefaction curves were constructed to visualize the level of diversity coverage in each sample, while to estimate alpha and beta diversity (Bray–Curtis and Jaccard) metrics, the qiime diversity core-metrics command implemented in QIIME2 software was used. Prior to this, samples were homogenized at the sampling depth level at 10,000 and 20,000 sequences per sample. Those samples that did not reach this level of sampling depth were discarded from the analyses. Finally, the taxonomic composition and relative abundance of phytoplankton per sample and analysis factor of the study can be visualized through the QiimeView interactive platform (https://view.qiime2.org/) and Pyloseq package v1.54.0 [33] in Rstudio v2025.05.1+513.

### 2.4. Diversity and Relative Abundance

Alpha-diversity metrics were computed in R (v4.5) using the phyloseq package. Prior to diversity estimation, ASVs with very low abundance (total counts < 10) or low prevalence (<2 samples) were removed, and samples with extremely low read depth were excluded. To minimize the influence of uneven sequencing effort, the ASV table was rarefied to the minimum library size using rarefy_even_depth (rngseed = 123, trimOTUs = TRUE). For alpha diversity groups differences (e.g., areas) were evaluated using Kruskal–Wallis tests followed by Benjamini–Hochberg–corrected pairwise Dunn’s tests. All computations were performed in R using the packages phyloseq, vegan, and rstatix.

Relative abundance analyses were performed using the same ASV table after normalization by total reads per sample. For each station, the abundance of each ASV was divided by the sum of reads in the corresponding sample, yielding proportional values (ranging from 0 to 1). Only ASVs taxonomically assigned to harmful species were retained for visualization. These normalized values were aggregated by sampling site and represented as bubble plots, where point size denotes the relative abundance of each ASV and color indicates species-level or clade assignment. All figures were produced in R using ggplot2 and tidyverse packages.

### 2.5. Environmental Parameters

Temperature and salinity averages were calculated using data from the South-Austral Operational Model (MOSA) to characterize the average pattern. MOSA is a numerical alternative to complement available regional oceanographic information in the Chilean Inland Sea [34] developed by Instituto de Fomento Pesquero (www.ifop.cl). MOSA has been in operation since 2017, with daily delivery of 3-day oceanographic forecasts. These forecasts are freely accessible on the CHONOS web portal [35]. For this study, we integrated the water column between the surface and 20-m depth during September 2020 and January 2021.

### 2.6. Nutrient Analysis

We quantified four nutrients central to primary production—nitrite (NO_2_^−^), nitrate (NO_3_^−^), phosphate (PO_4_^3−^), and silicate (SiO_2_)—to characterize water-column conditions influencing phytoplankton structure. At each station (Supplementary Data), we collected water from subsurface (0–20 m). Samples were filtered (0.45 µm), transferred to 5-mL high-density polypropylene vials, and frozen at −20 °C until analysis. Nutrients were measured on a segmented-flow autoanalyzer (QuAAtro39, Seal Analytical, Mequon, WI, USA) following standardized methods (Q-054-04 Rev.2 for NO_2_^−^, Q-119-11 Rev.2 for NO_3_^−^, Q-048-04 Rev.3 for PO_4_^3−^, Q-050-04 Rev.2 for SiO_2_). Nitrite was determined by diazotization of sulfanilamide under acidic conditions and coupling with N-(1-naphthyl)ethylenediamine dihydrochloride (NEDD), with absorbance at 550 nm; calibration with sodium nitrite standards (0–28 µg L^−1^ N) was linear (r ≥ 0.999) and the MDL was 0.1 µg L^−1^ N (EPA pt.136 app. B). Nitrate + nitrite (NO_3_^−^ + NO_2_^−^) was quantified after reducing nitrate to nitrite in a copper–cadmium coil at pH8 and applying the same colorimetric reaction; potassium nitrate standards (0–140 µg L^−1^ N) yielded r ≥ 0.999 and an MDL of 0.3 µg L^−1^ N. Phosphate was measured as the phosphomolybdenum blue complex in the presence of antimony and reduced with ascorbic acid (880 nm); calibration with KH_2_PO_4_ (0–124 µg L^−1^ P) produced r ≥ 0.999 and an MDL of 0.2 µg L^−1^ P. Silicate was quantified via reduction of the silicomolybdate complex with ascorbic acid, using oxalic acid to suppress phosphate interference (820 nm); sodium metasilicate nonahydrate standards (0–1.2 mg L^−1^ SiO_2_) were linear (r ≥ 0.999) with an MDL of 0.4 µg L^−1^ SiO_2_. This workflow provided precise and comparable nutrient measurements across stations, facilitating direct linkage between chemical gradients and phytoplankton community patterns (Yarimizu et al. [26]).

### 2.7. Ordination Analysis

To explore patterns in phytoplankton community structure, we performed a Principal Component Analysis (PCA) based on the abundance of amplicon sequence variants (ASVs) assigned to the classes *Bacillariophyceae* and *Dinophyceae*. Prior to the analysis, the ASV table was filtered to retain only samples with non-zero counts, and abundances were transformed to relative values per sample. To account for the compositional nature of the data and to reduce the influence of highly abundant taxa, we applied a Hellinger transformation to the relative abundance matrix.

Ordinations were conducted with the rda function implemented in the vegan R package (v.2.6-4), which is equivalent to a PCA using Euclidean distances on Hellinger-transformed data. Environmental variables (nitrite, nitrate, phosphate, silicate, temperature, salinity, oxygen, and fluorescence) were fitted post hoc onto the ordination using the envfit procedure (999 permutations) to evaluate their correlation with the ordination axes. Sample scores were plotted with 95% confidence ellipses drawn around centroids of each region to visualize the multivariate dispersion among geographical groups. All analyses were performed in R (v.4.3.3) using the phyloseq and vegan packages.

### 2.8. Phylogenetic Analyses

We performed all primary taxonomic assignments with the PR2 database, and applied the phylogenetic workflow only to ASVs from genera with low PR2 assignment confidence that also include known HAB-forming species (e.g., *Dinophysis*, *Alexandrium*). ASVs from Bacillariophyceae and Dinophyceae were aligned with MAFFT v7 to ensure positional homology [36]. Phylogenies were inferred in IQ-TREE2 with ModelFinder for automatic model selection under BIC [37,38] and node support was evaluated with 1000 ultrafast bootstrap (UFBoot) and 1000 SH-aLRT replicates [39,40]. Trees were rooted with predefined outgroups following standard conventions [41], and rooted outputs (.rooted.contree) were used for downstream labeling. To refine species-level names specifically for these PR2-limited, HAB-relevant genera, we annotated terminal tips via restricted BLASTn against the NCBI nt database (https://blast.ncbi.nlm.nih.gov/Blast.cgi?PROGRAM=blastn&PAGE_TYPE=BlastSearch&LINK_LOC=blasthome, accessed on 5 September 2025) constrained to the focal genus (-entrez_query “Genus [Organism]”), retaining for each ASV the top hit by bitscore and sequence identity and exporting summaries to _blast_all.tsv and _blast_top1.tsv. Trees were rendered in ETE3 [42] with binomial tip abbreviations, support values displayed when UFBoot ≥ 70%; we produced full phylograms (all ASVs shown as species-colored circles) and collapsed trees when monophyletic clades contained ≥ 2 ASVs of the same species.

## 3. Result

### 3.1. Diversity and Relative Abundance

**Figure 2:**
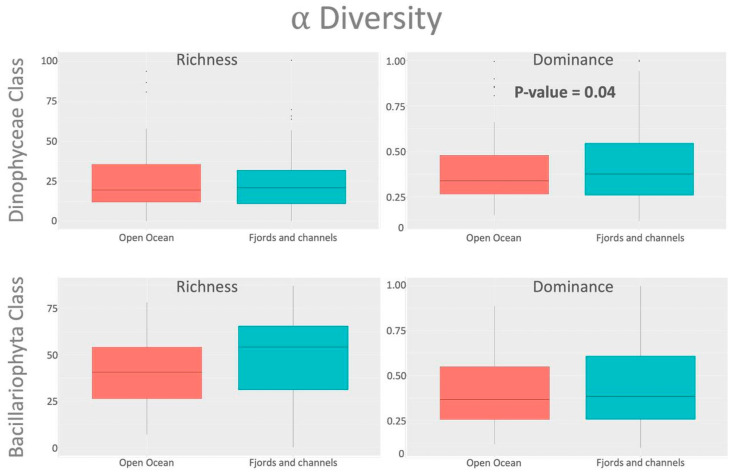
Alpha diversity for the Dinophyceae and Bacillariophyceae classes in phytoplanktonic communities from Open Pacific and Fjords/Channel areas.

A total of 3443 ASVs (Amplicon Sequence Variants) were identified, of which 26.9% were unique to the open ocean, 53.3% were unique to the fjord zone, and 19.8% were shared between both areas. For the Bacillariophyceae class, richness and dominance showed a non-significant variation between both areas (PERMANOVA, *p*-value > 0.05). Higher diversity for the fjord zone was observed compared to the open ocean (Figure 2). However, significant differences (PERMANOVA, *p*-value < 0.05) in alpha diversity between fjords and open oceans were detected for the Dinophyceae class considering dominance (Figure 2), while non-significant differences in ASV numbers (richness) were reported (PERMANOVA, *p*-value > 0.05).

**Figure 3:**
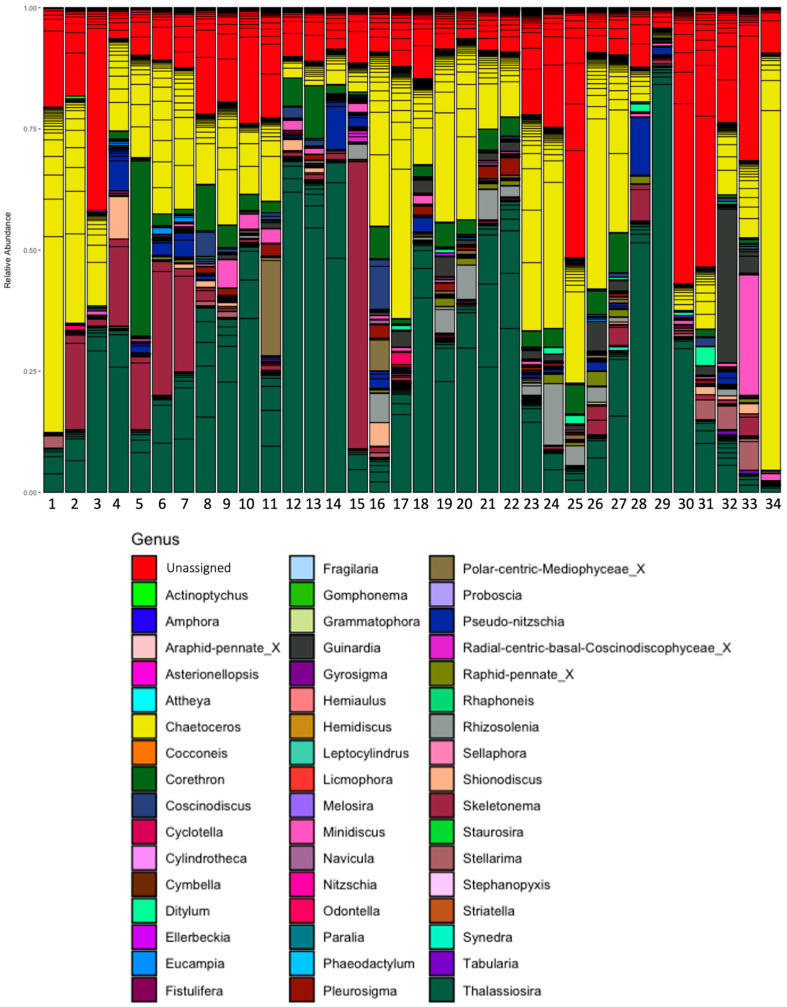
Relative abundance of diatoms in phytoplanktonic communities from Open Pacific (from 1 to 12) and Fjords/Channels (from 13 to 23) areas.

For diatoms (Figure 3), the genera *Thalassiosira*, *Chaetoceros*, *Skeletonema,* and *Pseudo-nitzschia* had the highest relative abundance across the studied sites. Around 25% of the relative abundance per site was archived to ASVs without taxonomic assignment at the order level (Figure 3).

**Figure 4:**
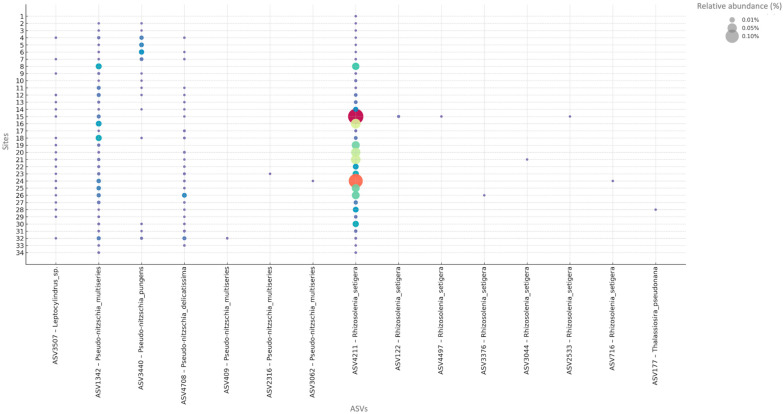
Relative abundance of selected ASVs across sampling stations for diatoms. Bubble plots show the relative abundance (>0) of each ASVs, ordered by genus along the x-axis and by sampling station (1–34) along the y-axis. Bubble size is proportional to the relative abundance of each ASV within each station, and bubble color encodes relative abundance, following a continuous gradient where colors change in 0.01% increments. ASV labels include the species name when available, while unlabeled ASVs remain at the genus or higher taxonomic level.

For diatoms, species-level ASVs displayed two contrasting distributional patterns. A first set was broadly present across the study region, including *Leptocylindrus* sp., *Pseudo-nitzschia delicatissima*, *Pseudo-nitzschia multiseries* (ASV1342), *Pseudo-nitzschia pungens* (ASV3440), and *Rhizosolenia setigera* (ASV4211). A second set showed more restricted, station-specific occurrences, notably *P. multiseries* (ASV2316, ASV3062, ASV409), *R. setigera* (ASV122, ASV2533, ASV3044, ASV3376, ASV4497, ASV716), and *Thalassiosira pseudonana* (ASV177). These patterns are captured in the bubble plots (Figure 4), where symbol size summarizes relative abundance by station and depth.

Within *Chaetoceros*, we detected a diverse set of ASVs, a subset of which resolved to species—primarily *C. cinctus*, *C. decipiens*, *C. diadema*, *C. muellerii*, and *C. pumilum*. Some of these ASVs were distributed throughout the sampled gradient, whereas others were concentrated in the fjords-and-channels sector. In contrast, ASVs assigned only to genus exhibited, overall, a broader geographic footprint than those resolved to species, consistent with multiple closely related lineages contributing diffuse abundance signals (Supplementary Figure S1).

**Figure 5:**
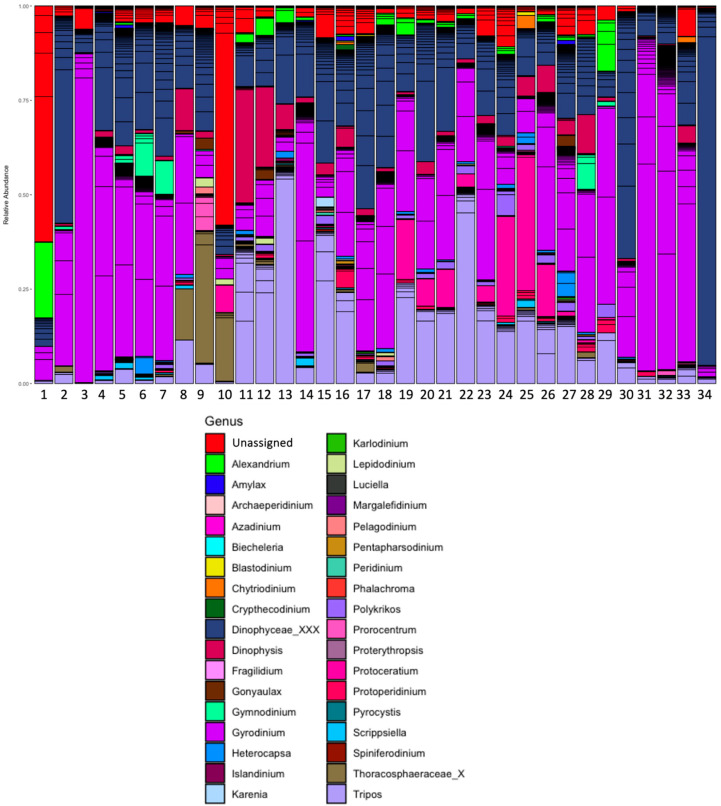
Relative abundance of genera Dinophyceae classes in phytoplanktonic communities from Open Pacific (from 1 to 12) and Fjords/Channels (from 13 to 23) areas.

In the Dinophyceae class (Figure 5) a high proportion of the relative abundance per site (25 to 50%) was represented by ASVs with taxonomic assignments that only reach the order level. At the genus level, though, *Gyrodinium*, *Tripos*, *Dinophysis*, *Protoceratium*, *Alexandrium*, *Gymnodinium*, and *Prorocentrum* had the highest relative abundances.

**Figure 6:**
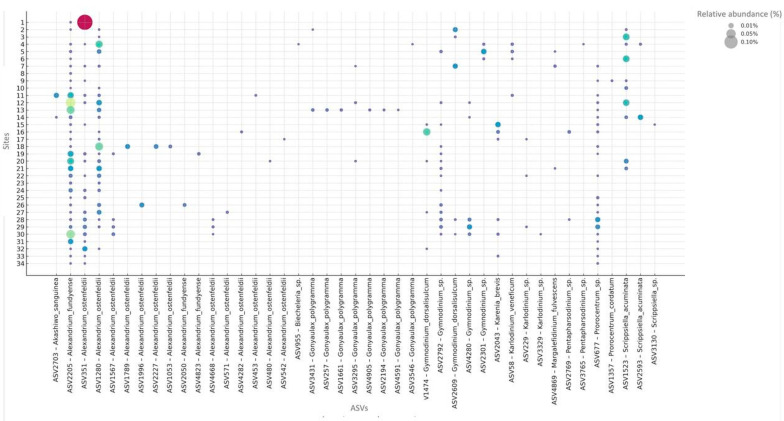
Relative abundance of selected ASVs across sampling stations for Dinophyceae class. Bubble plots show the relative abundance (>0) of each ASVs, ordered by genus along the x-axis and by sampling station (1–34) along the y-axis. Bubble size is proportional to the relative abundance of each ASV within each station, and bubble color encodes relative abundance, following a continuous gradient where colors change in 0.01% increments. ASV labels include the species name when available, while unlabeled ASVs remain at the genus or higher taxonomic level.

For Dinophyceae, we detected ASVs corresponding to the principal HAB-forming lineages reported for Chile (Figure 6), as well as taxa not previously described for the region. Within *Alexandrium*, 16 ASVs were recovered. Three ASVs matched *A. fundyense* (Formally *A. catenella*), with ASV2205 showing widespread relative abundance across most stations, whereas ASV2050 and ASV4823 were confined to specific localities. Two ASVs assigned to *A. ostenfeldii* (ASV1280, ASV351) displayed the broadest spatial ranges—ASV351 reaching the highest relative abundances—while the remaining *Alexandrium* ASVs (ASV1053, ASV1567, ASV1789, ASV1996, ASV2227, ASV4282, ASV453, ASV4668, ASV480, ASV542, ASV571) were patchy and consistently low.

Patterns in other Gymnodiniales paralleled those of *Alexandrium*. ASVs assigned to *Gymnodinium* at genus level (ASV2792, ASV2301, ASV4280) were geographically partitioned: ASV2792 occurred from Los Lagos southward, ASV2301 was restricted to Los Ríos, and ASV4280 appeared only at a few southern sites. Two *G. dorsalisulcum* ASVs (ASV1474, ASV2609) were likewise limited to Los Lagos and points south. *Karlodinium veneficum* was detected at four exposed-ocean stations, and two *Karlodinium*-only ASVs were confined to the Los Lagos–south sector. For *Gonyaulax polygrama*, seven of eight ASVs were restricted to exposed-ocean stations from Los Ríos northward; the exception, ASV3295, was recorded at a Chiloé site. Additional dinoflagellates occurred at lower frequencies and in localized patches, including *Akashiwo sanguinea* (ASV2703), *Biecheleria* sp. (ASV955), *Margalefidinium fulvescens* (ASV4869), and *Scrippsiella acuminata* (ASV1523, ASV2593). *Prorocentrum cordatum* (ASV1357) co-occurred with a more broadly distributed *Prorocentrum* sp. lineage (ASV677) (Figure 6).

Two diverse complexes dominate the Supplementary Materials. We identified 58 ASVs assigned to *Protoceratium reticulatum*: most were concentrated in fjords and channels, with the exception of ASV288, which was detected at nearly all stations and peaked at site 31 (Supplementary Figure S2). In parallel, 135 *Dinophysis* ASVs (none resolved to species) showed a distribution broadly similar to *P. reticulatum* but extending farther north from station 11 (northern Chiloé). Notably, ASV3479 was continuous across the survey area and reached its highest relative abundance at locality 30, where many *Dinophysis* ASVs co-occurred (Supplementary Figure S3).

### 3.2. Environmental Parameters and Nutrient Analysis

**Figure 7:**
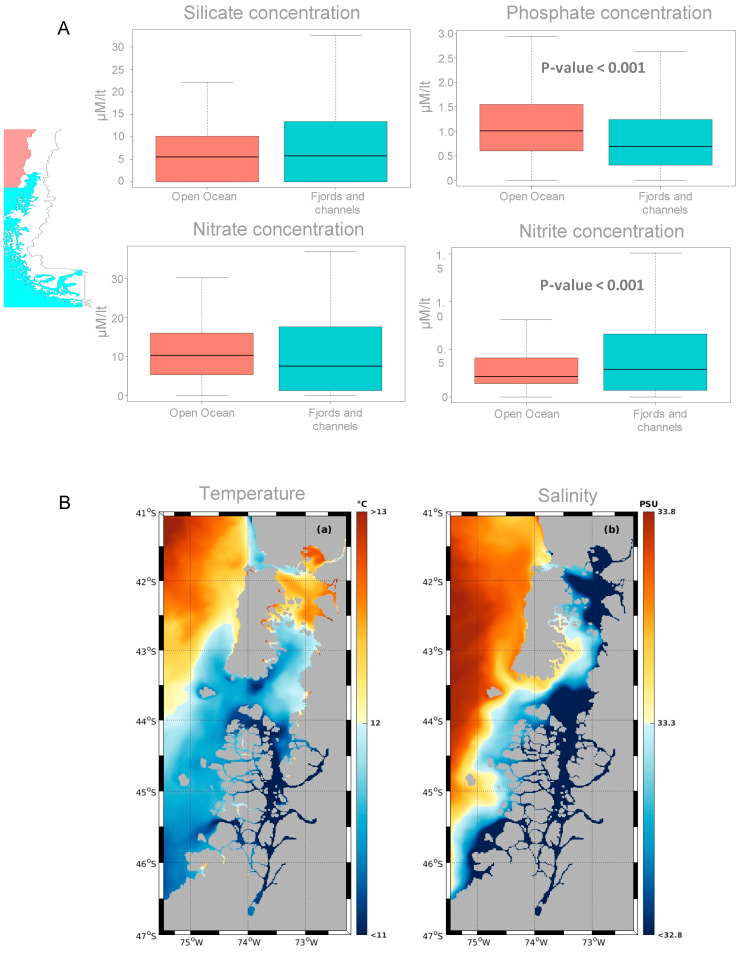
Environmental parameters quantified for each studied geographic area. (**A**) Nutrient concentrations (µM) of silicate, phosphate, nitrate, nitrite and nitrate for samples from both areas; light red and light blue denote Open Pacific and Fjords/Channels, respectively. (**B**) Integrated average temperature and salinity between surface and 20-m depth during September 2020 and January 2021, obtained from the MOSA operational model (http://chonos.ifop.cl/, accessed on 5 May 2023).

In the nutrient comparison (Figure 7A), silicate showed broadly similar distributions between the open ocean and fjords/channels, with the latter exhibiting a wider range and higher maxima but no clear statistical difference. Nitrate tended to be higher and more variable in fjords/channels, although not significantly so. By contrast, nitrite was significantly higher in fjords/channels (*p* < 0.001), whereas phosphate was significantly higher in the open ocean (*p* < 0.001). Together, these patterns indicate greater availability—and variability—of oxidized nitrogen forms in fjord environments, while phosphate is more abundant and comparatively stable offshore.

Surface fields from MOSA (Figure 7B) show clear hydrographic gradients between areas: temperature is highest offshore in the north (>13 °C) and decreases toward the inner fjords and the south (<11–12 °C), while salinity is also higher offshore (~33.5–33.8 PSU) and markedly reduced inside fjords and channels (~32.8–33.3 PSU), consistent with strong estuarine freshwater influence. Pronounced cross-shelf transitions at fjord mouths indicate mixing fronts. Overall, the fjord system is cooler and fresher than the adjacent open ocean, in line with the significant between-area differences reported above.

### 3.3. Ordination Analysis

**Figure 8:**
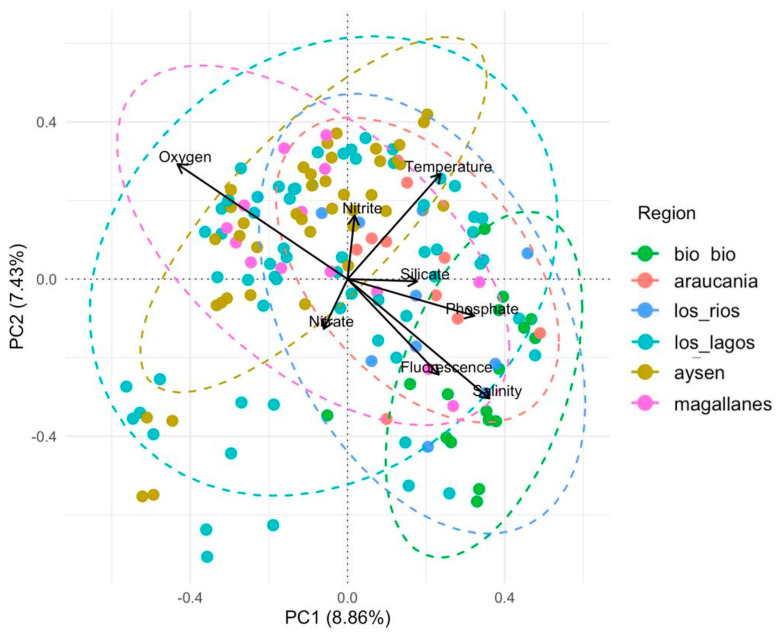
Principal Component Analysis (PCA) of phytoplankton ASV communities. Principal Component Analysis (PCA) based on Hellinger-transformed relative abundances of ASVs from diatoms and dinoflagellates. Each point represents a sampling station, colored according to geographic region, with 95% confidence ellipses showing the grouping pattern within each region. Environmental vectors (fitted using the envfit function) indicate variables significantly correlated with the ordination (*p* < 0.05). Salinity and phosphate explained the clustering of stations from the Biobío region, while temperature, nitrite, and dissolved oxygen were associated with the distribution of stations from the Aysén region.

The Principal Component Analysis (PCA) applied to Hellinger-transformed relative abundances of diatom and dinoflagellate ASVs did not reveal a clear separation among regions: point clouds and 95% confidence ellipses largely overlapped (Figure 8). The first two axes explained 8.86% (PC1) and 7.43% (PC2) of the total variance. PC1 represented an environmental gradient mainly associated with salinity, phosphate, and oxygen, whereas PC2 reflected variations related to temperature, nitrite, and nitrate. Samples from the Biobío region were grouped toward the positive end of PC1, where salinity and phosphate exerted the greatest influence, while those from Aysén were positioned toward the upper part of the biplot, associated with higher temperature, nitrite, and oxygen values. In contrast, samples from Araucanía, Los Ríos, Los Lagos, and Magallanes showed broad overlap, indicating a gradual transition in phytoplankton assemblage composition along the latitudinal gradient. Finally, nitrite, nitrate, and silicate exhibited a weaker influence on the distribution of samples within the multivariate space, suggesting a more localized or secondary effect of these variables on assemblage structure.

### 3.4. Phylogeny

To refine PR2-based assignments and verify species identities within HAB-relevant lineages, we reconstructed phylogenies for five focal genera. Overall, tree resolution and evolutionary depth varied among groups and the analyses resolved the diversity and relationships of bloom-forming organisms across five focal genera. *Chaetoceros* phylogeny (Supplementary Figure S4) recovered a well-resolved tree comprising 194 terminal sequences (ASVs) and 190 internal nodes, with a maximum evolutionary depth of approximately 0.41 units. The topology was organized into multiple well-supported clades corresponding to recognized species of the genus, revealing both consistent monophyletic groupings and a few moderately supported internal relationships. Overall, the main *Chaetoceros* species clustered into well-defined groups with high bootstrap values (bootstrap > 70), allowing clear identification of most taxa. Clades associated with *C. debilis*, *C. contortus*, *C. similis* and *C. didymus* exhibited strong support (bootstrap > 86) and short, compact branches, indicating low intraspecific divergence among assigned ASVs. On the other hand, within these clusters, subclades showed high internal cohesion. Moreover, the clade recovered as *C. dichatoensis* showed a low support value (bootstrap = 36). The tree also revealed peripheral branches with moderate support (bootstrap = 70–80), associated with single ASVs or small subgroups not clearly integrated into the main clades. Several environmental/unspecified sequences—labeled *Mediophyceae* sp., uncultured diatom, uncultured eukaryote, and uncultured marine eukaryote—occur as short satellite clusters interspersed among named taxa; these tips are placed on longer internodes but retain moderate-to-high support (bootstrap = 79–99). A few peripheral branches with moderate support (bootstrap = 70–80) involve single ASVs or small subgroups that do not clearly join the principal species clades.

The phylogenetic reconstruction of *Pseudo-nitzschia* generated a rooted tree (Supplementary Figure S5) containing 15 terminal sequences (ASVs) and 14 internal nodes, reaching a maximum evolutionary depth of 0.07 units. Overall topology was weakly resolved with an average bootstrap support of ~46 and only one minor clade showing strong support (bootstrap = 100). The majority of ASVs were distributed across poorly supported branches, including sequences identified as *P. multiseries* (5 ASVs), *P. cuspidata* (3 ASVs), *P. pungens*, *P. hasleana*, and *P. delicatissima*. Several ASVs matched other taxa (*Fragilariopsis cylindrus*, *Nitzschia palea*) or environmental sequences (uncultured eukaryote) (Supplementary Figure S5).

Tree reconstruction of *Dinophysis* (Supplementary Figure S6) recovered a rooted tree with 113 terminal sequences (ASVs) and 106 internal nodes, reaching a maximum evolutionary depth of 0.11 units and exhibited a low-resolution topology, with all nodes showing bootstrap values < 75, indicating limited statistical support for internal branching. Despite the overall weak resolution, the tree reveals a clear pattern of genetic heterogeneity. Most of the analyzed sequences clustered into a broad, poorly resolved assemblage dominated by *D. acuminata*–like lineages (bootstrap = 12), forming numerous shallow branches with short internal nodes.

Tree reconstructions of *Alexandrium* produced a rooted tree with 18 terminal sequences (ASVs) and 17 internal nodes, reaching a maximum depth of 0.83 evolutionary distance units (Supplementary Figure S7). It revealed a strongly monophyletic grouping, with high bootstrap support at the basal and internal nodes (values ranging between 74–97). Overall, the topology resolved two principal clades within *Alexandrium*: *A. ostenfeldii* (bootstrap = 83) and the *A. tamarense* complex, Group I (bootstrap = 97). Three ASVs initially annotated in GenBank/NCBI as “*A. tamarense*” clustered within Group I; under the formal revision [43] John et al. (2014) these correspond to *A. fundyense*. However, in line with the Chilean HAB literature—where PSP outbreaks have been historically attributed to “*A. catenella*”—we hereafter refer to this lineage as *A. catenella* (≡Group I sensu = *A. fundyense*) [44,45]. *A. ostenfeldii* isolates displayed short terminal branches with variable internal support, whereas the Group I/*A. catenella* lineage formed compact, well-supported clusters.

Phylogenetic analysis of *Protoceratium* produced a rooted tree (Supplementary Figure S8) with 60 terminal sequences (ASVs) and 59 internal nodes, reaching a maximum depth of 0.25. The average bootstrap support was moderate (mean = 47.8), but several terminal clades of two to five ASVs showed strong support (bootstrap ≥ 85). Most sequences (54 ASVs) clustered within a compact assemblage corresponding to *P. reticulatum*, characterized by short terminal branches and low intraspecific divergence.

## 4. Discussion

The South Pacific coast—particularly its fjord and channel systems—does not lack historical HAB observations based on optical microscopy; rather, what is missing are broad-scale, genomics-enabled surveys that resolve assemblage structure across environmental gradients. In this context, a monitoring program that routinely couples molecular methodologies with conventional approaches becomes critical conservation infrastructure [16,46,47]. Our metabarcoding data address precisely this gap, documenting spatial differences in the diversity and relative abundance of potentially harmful dinoflagellates and diatoms across areas and sampling sites. This approach meets two widely recognized needs: (i) expanding Southern Hemisphere coverage by incorporating sequencing into local observing networks, and (ii) sustaining time series that integrate biogeochemical and community indicators—including molecular tools—in fjord and open-ocean environments where stratification, hypoxia, and water darkening accelerate ecological responses relevant to HAB dynamics [47,48,49]. Altogether, our results highlight the value of a surveillance framework that integrates metabarcoding with traditional oceanographic monitoring to close the South Pacific metabarcoding gap and prioritize fjord and channel areas, reinforcing international calls to maintain long-term programs that incorporate molecular tools for improved HAB detection, forecasting, and management. The presented data still need better sampling site representation, particularly in the region of Magallanes.

### 4.1. Spatial Diversity Patterns and Drivers

Across the two provinces—exposed Pacific versus fjords— assemblage composition diverged in ways consistent with hydrographic context: coastal stratification/mixing and nutrient regimes structured taxonomic turnover, as captured by the PCA (Figure 8), broadly in line with coastal–fjord contrasts reported for phytoplankton systems [19,34,50,51]. Against this backdrop, our ordination showed that stations in Los Lagos (Figure 1, sites 15 to 22) clustered chiefly along phosphate and salinity, reflecting the interplay between oceanic inputs and continental runoff that modulates nutrient availability and surface stratification. In Aysén (Figure 1, sites 23 to 30), by contrast, structure aligned with oxygen, nitrite, and temperature, indicating variability in water-column ventilation, remineralization intensity, and the thermal regime characteristic of fjords. Within this framework, silica—largely supplied by rivers and glacier/ice-cap melt, intensified in high-latitude fjords—remains the principal nutrient supporting diatom growth [52,53]. Although silica did not differ significantly between areas (ANOVA, *p* > 0.05), relatively higher fjord values could help sustain the observed Bacillariophyceae diversity. Inorganic nitrogen showed the opposite partitioning: nitrate and nitrite were higher in fjords (only nitrite significant; ANOVA, *p* < 0.05), whereas phosphate was significantly higher offshore (ANOVA, *p* < 0.05). Across the two provinces—exposed Pacific versus fjords—assemblage composition diverged consistently with hydrographic context: turbulence and rapid nutrient supply tended to favor diatoms, whereas low-mixing, stratified lenses and shifts in nitrogen forms favored dinoflagellates, in agreement with our PCA (Figure 8) and with observations from Patagonian Chile [19,51,54,55] and classic functional theory [56,57,58]. Potential aquaculture-derived inputs may further inject phosphorus and nitrogen [59], warranting local evaluation of their effects on assembly processes. Finally, bacteria act as central nutrient recyclers and, via symbioses with microalgae, may confer competitive advantages to harmful species; future work should explicitly integrate molecular bacterial diversity into these gradients [23].

Differences in primary productivity between fjords and the open ocean emerge from complex oceanographic processes that have selected phytoplankton over contemporary and geological time, yielding contrasting genetic fingerprints between South Pacific provinces, where temperature is a dominant structuring factor. In high-latitude ecosystems, warming can accelerate glacial melt and ice-shelf detachment, increasing the release of organic and inorganic forms of nitrogen, phosphorus, and silica, and altering microbial communities [60,61,62]. During our study period, temperature and salinity modeled with MOSA (http://chonos.ifop.cl/) differed significantly between areas (ANOVA, *p* < 0.05), consistent with the spatial segregation (PERMANOVA, *p* < 0.05) observed in the PCoA and with trends reported for Arctic fjords in small phytoflagellates [63]. Altogether, these patterns indicate that the detected differences in diversity and abundance are not random but arise from environmental filters consistent with microscopy-based literature and the functional ecology of dominant groups, reinforcing the value of integrating molecular diversity metrics with hydrographic gradients to anticipate bloom scenarios [64,65].

At a historical scale, our results fit within the documented spatiotemporal expansion of HABs in Chile, with increased geographic coverage and recurrence over six decades [66] (Barría et al., 2022). Rather than restating gradients, the axes identified above provide a mechanistic lens for why particular dinoflagellate and diatom clades emerge differentially across areas: the combination of surface stratification, inorganic nutrient availability (including nitrite), and water-column ventilation matches microscopy-based conditions that precede and accompany blooms in southern Chilean fjords. Thus, metabarcoding not only captures the fine taxonomic turnover underlying those historical series, but also links specific lineages to local drivers—key for moving from description to operational forecasting and adaptive management [67,68]. Sustained observations will help distinguish signals of change (e.g., warming, increased continental inputs, or anthropogenic pressures) from natural variability, strengthening monitoring programs that integrate molecular tools with conventional observations [48].

### 4.2. Detection of HABs Species

We detected ASVs corresponding to multiple taxa historically linked to HABs in Chile, including *Alexandrium* species, *Dinophysis* spp., and *Pseudo-nitzschia* spp., together with *Karenia* spp. reported in fish-kill events (1999, 2017) and in recent isolates of *K. selliformis* from Patagonia (Chile) [8,45,66,69,70,71,72,73]. Notably, we observed a metabarcoding signal assigned to *Karenia brevis*, a species not formally documented for Chilean waters, where *Karenia* events are attributed primarily to *K. selliformis* (and occasionally other *Karenia* spp.) [71,73]. Given that *K. brevis* is typically associated with red tides in northern oceans, this detection warrants targeted verification (e.g., microscopy or species-specific qPCR primers) and is consistent with plausible introduction pathways such as ballast water, documented for *Karenia* spp. elsewhere [74,75].

Beyond these taxa, we also detected ASVs for *Leptocylindrus* spp., *Thalassiosira pseudonana*, *Akashiwo sanguinea*, *Biecheleria* sp., *Gymnodinium* spp., *Karlodinium veneficum*, and *Protoceratium reticulatum*. Their distributions help explain the contrasting assemblages between fjords and the exposed Pacific. Broadly euryhaline/eurythermal diatoms such as *T. pseudonana* and the coastal chain-forming *Leptocylindrus* typically tolerate strong salinity and temperature gradients and therefore appeared in both provinces [76,77]. By contrast, several dinoflagellates linked to low-mixing, stratified, nutrient-replete surface lenses characteristic of fjords—e.g., *Gymnodinium* (brown-tide events reported for Magellanic fjords), *K. veneficum* (karlotoxin-producing, often favored in mesohaline/stratified waters), and *P. reticulatum* (YTX-producer recurrent along Patagonian fjords)—were enriched or exclusive to fjord stations [69,78,79]. A. sanguinea, a mixotrophic, ichthyotoxic species with contact-mediated effects, and *Biecheleria* spp., reported as euryhaline/eurythermal, were likewise more frequent where surface stability and nutrient recycling are enhanced; occasional presence offshore is consistent with advection from coastal sources and broad physiological tolerances [80,81]. Altogether, taxa occurring in both areas (e.g., *Leptocylindrus*, *T. pseudonana*) likely reflect wide niches and connectivity, whereas fjord-restricted occurrences (e.g., *Gymnodinium*, *K. veneficum*, *P. reticulatum*) align with local stratification, nitrite/oxygen patterns, and potential anthropogenic nutrient inputs documented for these systems—mechanistic filters that match our PCA structure and the known HAB ecology of Patagonian fjords.

In our results, using the PR2 curated database, these ASVs were identified as *A. fundyense* (Group I). Following the formal revision by John et al. (2014) [43], and to align with Chilean HAB monitoring programs, we hereafter refer to this Group I lineage as *A. catenella*. Because the short 18S region cannot reliably discriminate among Groups I–V, we therefore report these records as *A. catenella* (tamarense complex; Group I) throughout the text. Within this framework, we found no evidence for other members of the complex (e.g., *A. pacificum*) in our data. This nomenclatural choice (Group I ≡ *A. fundyense* sensu; historical usage in Chile = *A. catenella*) avoids ambiguity around “*A. tamarense*” and aligns our reporting with current taxonomy and national usage.

Beyond these detections, results should be interpreted in light of metabarcoding limitations. Taxonomic resolution depends on the marker: 18S (V4/V9) offers broad coverage but often resolves only to genus in cryptic complexes, whereas LSU (D1–D3) or ITS better discriminate species at the cost of reduced universality [82,83]. Multi-marker designs (e.g., 18S + LSU/ITS) reduce primer-mismatch bias and increase species recovery relative to single-amplicon approaches [84,85]. Quantitatively, reads ≠ biomass/abundance because rDNA copy-number variation, amplicon length, and PCR bias can skew relative counts; thus, relative abundances should be treated as compositional indicators and, when critical, validated with species-specific qPCR or microscopy counts [86,87]. A further constraint is the geographic coverage of reference databases: incompleteness and regional biases can lead to non-assignments or misassignments, disproportionately affecting underrepresented regions such as the South Pacific [88,89]. Although resources like PR2 and 18S primer catalogs have improved standardization, gaps persist for many marine protists [32,90]. It is therefore imperative to build regional networks (institutes, monitoring programs, and reference collections) that generate and deposit voucher-backed sequences from South Pacific biota in curated repositories (PR2/SILVA), ensuring that local diversity is represented and improving the fidelity of taxonomic assignment. Integrated strategies—metabarcoding for broad surveillance and early detection, alternative markers + qPCR for species-level confirmation, and microscopy counts to anchor quantitative interpretation—maximize diagnostic and forecasting power, especially for complexes such as *Alexandrium* or *Karenia* [91,92].

### 4.3. Phylogeny

Harmful microalgal bloom (HAB) species—including diatoms (*Chaetoceros*, *Pseudo-nitzschia*) and dinoflagellates (*Dinophysis*, *Protoceratium*, and *Alexandrium*)—are key components of marine phytoplankton communities and play central roles in the ecology of coastal and fjord systems in southern Chile, where oceanic and estuarine dynamics converge [4,93]. Understanding their evolutionary structure is fundamental for linking phylogenetic diversity to bloom formation, toxin production, and environmental adaptation. The phylogenetic analyses presented here reveal both strong species-level monophyly and heterogeneous internal topologies among genera, suggesting that evolutionary diversification in these HAB taxa is driven by a combination of ecological specialization, restricted dispersal, and the intrinsic limitations of ribosomal markers in metabarcoding studies.

The *Chaetoceros* phylogeny displayed high species-level resolution, with well-supported clades corresponding to *C. debilis*, *C. contortus*, *C. similis* and *C. didymus*, each showing strong bootstrap values (bootstrap > 86) and short, compact branches (Supplementary Figure S4). Such topologies suggest genetically stable and recently diversified lineages, possibly maintained by recurrent bloom events and environmental selection in coastal waters [94,95,96]. Conversely, a low support value (bootstrap = 36) was recovered in the *C. dichatoensis* clade, reflecting limited resolution of the marker and possible intraspecific heterogeneity. The close clustering of ASVs within each species indicates low intraspecific divergence, consistent with the clonal reproduction and seasonal recurrence typical of *Chaetoceros* populations. The presence of several peripheral branches with moderate support (bootstrap = 70–80) and long internodes, often associated with single ASVs or small environmental subgroups, suggests the occurrence of rare ribotypes or undersampled diversity within the genus [96]. Similar patterns of genetic coherence combined with peripheral variability have been described in molecular studies of *Chaetoceros* species from temperate regions, where environmental gradients and hydrodynamic isolation promote fine-scale differentiation [97,98]. These results reinforce that *Chaetoceros* exhibits strong phylogenetic structuring at the species level, but also subtle intra-lineage microdiversity that may represent early stages of ecological or geographic divergence [96,99].

The phylogenetic reconstruction of *Pseudo-nitzschia* (Supplementary Figure S5) recovered 15 ASVs in a compact but weakly resolved tree (average bootstrap ~46). Expected complexes (*delicatissima* and *seriata*) were not retrieved, and sequences of *P. multiseries*, *P. cuspidata*, *P. delicatissima*, *P. pungens*, and *P. hasleana* appeared interspersed with non-target taxa (*Fragilariopsis*, *Nitzschia*), reflecting both the limited resolution of the 18S rRNA marker and the heterogeneous nature of the dataset [99,100].

The phylogenetic reconstruction of *Dinophysis* (Supplementary Figure S6) revealed a low-resolution topology dominated by *D. acuminata*–like lineages, consistent with the extensive genetic heterogeneity previously reported within this morphospecies complex [101]. The overall low bootstrap values (average = 58) suggest limited phylogenetic resolution at the 18S rRNA locus, reflecting the high degree of sequence conservation characteristic of this marker in dinoflagellates [102]. Nonetheless, the broad assemblage observed indicates that *D. acuminata* represents a species complex encompassing multiple cryptic or semi-cryptic lineages that are morphologically indistinguishable but genetically diverse.

The *Alexandrium* phylogeny (Supplementary Figure S7) recovered two well-defined clusters corresponding to *A. ostenfeldii* (13 ASVs) and *A. catenella* (3 ASVs, bootstrap = 97). Although bootstrap support across deeper nodes was generally low (average ~49), the strong support of the *A. catenella* clade highlights its genetic distinctiveness within the dataset. Our three Alexandrium ASVs that matched *A. catenella,* for which the current and widely adopted nomenclature recognizes the valid binomen for Group I lineages [43]. Given the limited discriminatory power of the short 18S region to resolve Groups I–V within the complex, we report these records as *A. catenella* (tamarense complex), aligning with the prevailing usage in the Chilean HAB literature and monitoring programs.

The dominance of *Protoceratium reticulatum* sequences in phylogeny (Supplementary Figure S8) reflects the ecological prevalence of this dinoflagellate in temperate and subpolar coastal waters, where it is a recurrent component of planktonic assemblages and a known producer of yessotoxins [103]. The compact and low-divergence clustering of most ASVs suggests limited intraspecific variability at the 18S rRNA locus, consistent with earlier reports that this marker provides poor resolution within *Protoceratium* and related dinoflagellates [102,104]. The results support the view that *P. reticulatum* forms a genetically cohesive lineage, but higher-resolution markers such as LSU rDNA or ITS are required to investigate population structure and cryptic diversity within this taxon [43].

Overall, the phylogenetic patterns recovered across diatoms (*Chaetoceros*, *Pseudo-nitzschia*) and dinoflagellates (*Dinophysis*, *Protoceratium*, *Alexandrium*) highlight a complex mosaic of evolutionary processes shaping harmful algal bloom (HAB) diversity in southern Chile. The coexistence of strongly supported monophyletic clades and unresolved or shallowly branching topologies reflects contrasting evolutionary trajectories among genera, likely driven by ecological specialization, geographic isolation, and reproductive strategies. Despite the conservative nature of the 18S rRNA marker, which limits its ability to discriminate among closely related or cryptic species [105,106], the results demonstrate its robustness for establishing higher-level phylogenetic structure and revealing patterns of recent diversification within HAB taxa. The detection of cohesive, low-divergence lineages in *Chaetoceros* and *Protoceratium*, contrasted with heterogeneous assemblages in *Dinophysis* and *Pseudo-nitzschia*, suggests that selective pressures linked to fjord–ocean environmental gradients, such as salinity, stratification, and nutrient fluxes, contribute to the maintenance of genetic structure and local adaptation. From an applied perspective, these findings underscore the need to integrate high-resolution molecular markers (e.g., ITS, LSU, and genomic barcodes) into Chile’s national HAB monitoring programs to improve species delimitation, track the spread of toxigenic lineages, and refine early-warning models for bloom events. Understanding the evolutionary context of bloom-forming microalgae thus represents not only a fundamental step toward accurate taxonomy and biodiversity assessment but also a critical component for predicting and mitigating the ecological and socio-economic impacts of harmful algal blooms in Chilean coastal ecosystems.

## 5. Conclusions

Our metabarcoding survey provides a regional baseline of phytoplankton molecular diversity across two contrasting oceanographic provinces of southern Chile—the stratified, freshwater-influenced fjords/channels and the more oceanic open Pacific. Community structure differed between provinces and aligned with environmental gradients: fjords/channels were associated with lower salinity and higher phosphate, whereas the open Pacific aligned with higher temperature, nitrite, and dissolved oxygen (PCA on Hellinger-transformed data with envfit). Beyond genus-level patterns, phylogeny-aided assignments documented several HAB-linked species previously reported for the region and revealed additional putative species, with station-wise relative abundances summarized in the main figures and supplements. Together, these results identify core and diagnostic lineages for each province and highlight environmental drivers that may shape their distribution. While short 18S markers limit universal species-level resolution, the province-level contrasts are robust across taxonomic ranks and provide actionable context for surveillance. Future work integrating quantitative microscopy, higher-resolution loci (e.g., LSU D1–D2) and seasonal sampling will refine species confirmation, phenology, and environmental thresholds relevant to HAB risk along the Southeast Pacific coast.

## Data Availability

The original contributions presented in the study are included in the article/Supplementary Material, further inquiries can be directed to the corresponding author.

## Supplementary Materials

The following supporting information can be downloaded at: https://www.mdpi.com/article/10.3390/microorganisms13122746/s1, Figure S1: Relative abundance of *Chaetoceros ASVs* assigned just a genus level. Figure S2: Relative abundance of *Protoceratium reticulatum ASVs.* Figure S3: Relative abundance of *Dinophysis ASVs* assigned just a genus level. Figure S4: *Chaetoceros* phylogeny based on study ASVs. Node support is shown when UFBoot ≥ 70 as SH-aLRT/UFBoot (×100). Tips represent ASVs (circles) and references; colors denote species or clade (legend at right). Figure S5: *Pseudo-nitzschia* phylogeny based on study ASVs. Tips represent ASVs (circles) and references; colors denote species or clade (legend at right). Node support (SH-aLRT/UFBoot ×100) is shown when UFBoot ≥ 70. Figure S6: *Dinophysis* phylogeny based on study ASVs. The tree was rooted with predefined outgroups. Tips represent ASVs (circles) and references; colors denote species/clade (legend at right). Node support (SH-aLRT/UFBoot ×100) is shown when UFBoot ≥ 70. Figure S7: *Alexandrium* phylogeny based on study ASVs. Rooting used predefined outgroups (*Protoceratium reticulatum*, *Polyedra*). Tips represent ASVs (circles) and references; colors denote species/clade (legend at right). Node support is shown as SH-aLRT/UFBoot (×100) when UFBoot ≥ 70. Figure S8: *Protoceratium* phylogeny based on study ASVs. The tree was rooted with *Alexandrium ostenfeldii* as outgroup. Tips are ASVs (circles) and references; colors indicate species/clade (legend at right). Node support (SH-aLRT/UFBoot ×100) is shown when UFBoot ≥ 70.
