# Supplementary material for: Patagonian Fjords/Channels vs. Open Ocean: Phytoplankton Molecular Diversity on Southern Chilean Coast
Source: Microorganisms. 2025 Dec 2;13(12):2746. doi: 10.3390/microorganisms13122746 (PMC12736203; doi:10.3390/microorganisms13122746)
Supplement: Supplementary file 1 [file microorganisms-13-02746-s001.zip › Suplementary figures.pdf]

Supplementary Figure S1. Relative abundance of *Chaetoceros ASVs* assigned just a genus level.

Supplementary Figure S2. Relative abundance of *Protoceratium reticulatum ASVs*.

Supplementary Figure S3. Relative abundance of *Dinophysis ASVs* assigned just a genus level.

Supplementary Figure S4. *Chaetoceros* phylogeny based on study ASVs. Node support is shown when UFBoot ≥ 70 as SH-aLRT/UFBoot (×100). Tips represent ASVs (circles) and references; colors denote species or clade (legend at right).

Supplementary Figure S5. *Pseudo-nitzschia* phylogeny based on study ASVs. Tips represent ASVs (circles) and references; colors denote species or clade (legend at right). Node support (SH-aLRT/UFBoot ×100) is shown when UFBoot ≥ 70.

Supplementary Figure S6. *Dinophysis* phylogeny based on study ASVs. The tree was rooted with predefined outgroups. Tips represent ASVs (circles) and references; colors denote species/clade (legend at right). Node support (SH-aLRT/UFBoot ×100) is shown when UFBoot ≥ 70.

Supplementary Figure S7. *Alexandrium* phylogeny based on study ASVs**.** Rooting used predefined outgroups (*Protoceratium reticulatum*, *Polyedra*). Tips represent ASVs (circles) and references; colors denote species/clade (legend at right). Node support is shown as SH-aLRT/UFBoot (×100) when UFBoot ≥ 70.

Supplementary Figure S8. *Protoceratium* **phylogeny based on study ASVs.** The tree was rooted with *Alexandrium ostenfeldii* as outgroup. Tips are ASVs (circles) and references; colors indicate species/clade (legend at right). Node support (SH-aLRT/UFBoot ×100) is shown when UFBoot ≥ 70.
